# Retrieving Transgender and Gender Diverse Literature: Protocol for the Development and Validation of 2 Search Hedges

**DOI:** 10.2196/76055

**Published:** 2026-01-23

**Authors:** Chi Dinh, Zack Marshall, Scott Marsalis, Ryn Gagen, Preet Kang, Avery Everhart

**Affiliations:** 1 Department of Community Health Sciences Cumming School of Medicine University of Calgary Calgary, AB Canada; 2 School of Social Work McGill University Montreal, QC Canada; 3 O'Brien Institute for Public Health University of Calgary Calgary, AB Canada; 4 Sciences, Agriculture & Engineering University of Minnesota Libraries University of Minnesota Saint Paul, MN United States; 5 Health Sciences Library University of Minnesota Saint Paul, MN United States; 6 Faculty of Pharmaceutical Sciences University of British Columbia Vancouver, BC Canada; 7 Department of Geography Faculty of Arts University of British Columbia Vancouver, BC Canada

**Keywords:** evidence synthesis, database searching, search hedge validation, search filter validation, Two-Spirit, transgender, nonbinary, gender minority, information justice

## Abstract

**Background:**

Searching for transgender and gender diverse (TGD) references within large academic databases can be a challenging process, partly due to the dynamic and diverse definitions of words and terminologies used by multiple interest holders. Search hedges are preestablished search strings that aid in the efficacy of identifying and screening relevant articles. Validated search hedges focused on TGD people and topics will aid in identifying relevant literature.

**Objective:**

This study aims to develop and validate the sensitivity and precision of 2 interdisciplinary and cross-cultural TGD search hedges designed for retrieving references from MEDLINE and APA PsycInfo, both on the Ovid platform.

**Methods:**

Searches were conducted using the finalized search hedges via Ovid on June 7, 2024, yielding 31,055 references from MEDLINE and 22,924 references from APA PsycInfo. A random sample of 2330 records from MEDLINE and 2293 records from APA PsycInfo will be independently screened by at least 2 team members. At the title and abstract screening stage, references will be excluded if they (1) use solely binary terminology to describe gender, (2) focus on psychometric measurement of gender, or (3) focus on intersex or differences of sex development (DSD) topics. References will be included if they (1) report on transgender or gender diverse people, or both, in their sample; or (2) specifically discuss TGD communities or TGD topics. References without an abstract will be categorized as *No_Abstract.* References in which the TGD population is unclear will be categorized as *LGB_Maybe_T* or *Mixed_Topics.* Only references in the *No_Abstract*, *LGB_Maybe_T*, or *Mixed_Topics* categories will proceed to the full-text screening phase. In the full-text screening phase, references will be categorized as included if they (1) clearly distinguish between sexual identity and gender identity, (2) mention or discuss TGD topics or experiences in the Methods or Results sections, (3) communicate consideration for participants’ gender self-identification and experiences, or (4) consider TGD populations as a distinct subpopulation. The results of the screening process will be used to calculate precision and sensitivity, with a targeted sensitivity of 100% and a targeted precision of 76% for each search hedge.

**Results:**

Validation and data analysis are projected to be finished by December 2025, with results expected to be published in 2026.

**Conclusions:**

Rigorous and transparent knowledge synthesis processes, starting with a high-quality search hedge, can help inform and equip community members, clinicians, policymakers, and other key decision-makers with scientifically sound evidence.

**International Registered Report Identifier (IRRID):**

DERR1-10.2196/76055

## Introduction

### Background

Knowledge synthesis, also called evidence synthesis, is the process of identifying, evaluating, and critically integrating all available evidence on a topic [1]. This process is a foundational practice for the development of evidence-based decision-making [1]. Effective and comprehensive search strategies are one of the core components of knowledge synthesis [2]. To support robust search strategies, search hedges are increasingly used. A search hedge consists of pre-established search strings designed to comprehensively retrieve references in a topic area from one or multiple bibliographic databases [3]. A search hedge is typically developed by librarians working with subject matter experts and should be validated for its performance [4]. Validated search hedges can help ensure all relevant literature is retrieved, reduce the number of irrelevant articles screened, and increase the efficiency of resource use in the knowledge synthesis process.

In addition to hedges with a specific subject matter focus, more recent publications have emphasized other search attributes, such as geographic, methodological, or population-specific searches [5-7]. While search hedges focused on combined sexual and gender minority groups are available, there are currently no validated and published search hedges that focus on transgender and gender diverse (TGD) communities and topics [8,9]. In this protocol, we use the term *TGD* to describe people who do not identify with their assigned gender at birth. This definition has the potential to also describe intersex people and people with differences of sex development (DSDs), and there are indeed certain overlapping experiences between TGD and intersex people. However, we follow the recommendations of organizations such as interACT: Advocates for Intersex Youth who make plain that being intersex and being TGD are distinct categories of identity and experience. Thus, we will not include articles studying DSDs or intersex communities unless it is clear within the article that the study sample includes people who are both intersex and transgender to respect these identities and experiences as distinct from those of TGD communities.

Additionally, we recognize that *TGD* can be understood as an anglophone export or a category imposed onto autochthonous sex and gender systems that have been devalued, erased, or even criminalized through imperialism and settler colonialism [10,11]. Thus, our search hedges incorporate terminology beyond that centered within the medical literature of the anglophone world and Global North. This decision to incorporate culturally specific terms, while intended to be inclusive, also runs the risk of reproducing the centrality of not only English-language terms but also medicalized models of understanding gender nonconformity and gender diversity.

TGD people face systematic marginalization and oppression unique from those of the cisgender heterosexual population and their sexual minority peers [12,13]. Misconceptions and disinformation regarding the experiences, quality of life, and health of TGD people further fuel the discrimination against this population [14-16]. Rigorous and transparent knowledge synthesis processes, starting with high-quality search hedges, can help inform and equip community members, clinicians, policymakers, and other key decision-makers with scientifically sound evidence.

### Validation Structure

#### Overview

Developing structured search strategies focused on TGD populations is complex for several reasons, and our nonstandard screening process was developed to address these complexities. In addition to the *Include* and *Exclude* categories in a standard literature screening process, 2 additional categories were created for the title and abstract screening phase: *Mixed_Topics* and *LGB_Maybe_T*. The purpose of the *Mixed_Topics* category is to differentiate articles related to TGD people and topics from those related to cisgender people and topics. The purpose of the *LGB_Maybe_T* category is to differentiate articles on gender identity topics relevant to TGD people from those on Two-Spirit, lesbian, gay, bisexual, transgender, queer, intersex, asexual, or additional sexual and gender diverse (2SLGBTQIA+) topics.

#### Mixed_Topics Category

One challenge in identifying and retrieving literature relevant to TGD populations is the variations in context and usage of polysemic terms such as *gender diversity* or *gender inclusivity* (for a complete list of polysemic terms relevant to these hedges, see Multimedia Appendix 1). For example, the term *gender inclusivity* can be used to describe the inclusion of TGD people in historically underrepresented fields, but it can also be used to refer to the inclusion of cisgender women in fields and occupations historically dominated by men. Due to this usage in both cisgender and TGD contexts, inclusion of these terms in a search hedge has the potential to increase the number of nonrelevant references being retrieved, imposing additional burden on the subsequent literature screening process. However, exclusion of these terms risks compromising the comprehensiveness of the search hedges. Thus, the category *Mixed_Topics* was created to evaluate the accuracy and usefulness of including these polysemic terms.

#### LGB_Maybe_T Category

An additional challenge with searching for TGD literature is the conflation of gender identity and sexual orientation: authors often report gender identity inconsistently, if at all [17-19]. This lack of specificity means that gender minority populations are often homogenized within the broader 2SLGBTQIA+ acronym without differentiating the experiences of gender minority groups as distinct from those of sexual minority groups. While sexual and gender minority groups share close social and political struggles, and TGD people can also have a sexual minority identity (eg, a nonbinary bisexual individual), people with gender minority identities often experience unique forms of discrimination stemming from cisnormativity [20]. Therefore, articles that are relevant to 2SLGBTQIA+ populations are not guaranteed to be relevant to TGD populations, and articles that only use the term 2SLGBTQIA+ (or its variations) in their titles and abstracts do not provide sufficient information to determine their relevancy to TGD people and topics. The category *LGB_Maybe_T* was created to (1) confirm the relevance of articles using the acronym 2SLGBTQIA+ to the TGD population and (2) analyze patterns of usage of TGD terms in 2SLGBTQIA+ research.

### Precision and Sensitivity

Precision and sensitivity are 2 measurements commonly used in the validation process of search hedges and together offer quantifiable reference points in the performance of a hedge. Precision is the ability of a search hedge to minimize the retrieval of nonrelevant articles [21,22]. Precision is calculated as the proportion of relevant over total retrieved articles [22]. Sensitivity, also called relative recall, is the ability of a search hedge to retrieve all relevant articles [21,22]. Sensitivity is calculated as the proportion of articles from a gold-standard set that are retrieved by a search hedge [22]. The most common method of establishing a gold-standard set for the validation of a search hedge is through manually searching a journal, a set of journals, or a database [22]. However, the main disadvantage of this method is its high time consumption and low article yield [22]. Another approach to finding a gold standard is using a previously assembled collection [22]. In this study, the gold-standard set consists of 144 TGD articles from Knowsy (search year: 2019; for a full list of articles in the gold-standard set, see Multimedia Appendix 2). Knowsy is an online dataset of scoping reviews, systematic reviews, and structured reviews focused on 2SLGBTQIA+ topics [23,24]. Articles in Knowsy were identified by systematically searching 4 academic databases (MEDLINE, APA PsycInfo, Embase, and CINAHL) and selected in accordance with the standard 2-step screening process performed by 2 independent, trained reviewers [23,24]. Each reference in Knowsy has been labeled with standardized category tags for topics, populations, and type of knowledge synthesis by a trained reviewer, with independent validation by a second reviewer [23,24].

Special consideration also needs to be paid to ensure ethical and responsible research when working with a marginalized community, such as gender minority communities. Accountability and transparency in the search and research process are 2 key components to ensure that researchers do not further alienate already marginalized people. One of the best practices to accomplish these goals is the publication of a protocol at the start of the research process [25]. A protocol offers detailed background information on the search and screening process and increases the reproducibility of the results. It also provides an accountability mechanism as deviations from the protocol need to be identified when reporting study findings. As validations of search hedges are still uncommon, protocols for the validation of these hedges are even more limited [26-28]. The scant literature regarding search hedge validation methods could further discourage this type of research from being conducted as lack of established methods can increase the amount of time and resources dedicated to this process. Thus, publishing a search hedge validation protocol that includes details about the methods used aids in knowledge and resource sharing, as well as facilitating the peer review process and increasing research rigor. In this paper, we present a protocol for the validation of 2 TGD search hedges using a novel validation structure.

### Objectives

This protocol describes the methods for a search hedge validation. The objectives are to (1) validate 2 interdisciplinary and cross-cultural TGD search hedges designed for retrieving references in MEDLINE and APA PsycInfo, (2) determine the usage pattern of TGD polysemic terms in the literature, and (3) assess the usage pattern of TGD terms in 2SLGBTQIA+ literature.

## Methods

### Ethical Considerations

Ethics approval will not be sought for this study because it will only include analysis of published articles. This study will not involve human participants, medical records, patient information, observations of public behaviors, or secondary data analyses.

### Search Hedge Design

To develop and validate the 2 search hedges, we followed a 2-stage process: a preliminary search and a finalized search.

#### Stage 1: Preliminary Search Hedges

Two preliminary search hedges were developed in 2023 by 2 librarians with the goal of obtaining new insights into the effectiveness of the searches and inform sample size calculations for the full validation study. The MEDLINE search produced 49,796 results, and the APA PsycInfo search produced 38,344 references. References were imported into EPPI-Reviewer (EPPI-Centre) and randomly allocated into screening groups [29]. Two team members then screened 0.5% (239/49,796) of the references from MEDLINE and 0.5% (192/38,344) of the references from APA PsycInfo.

In terms of precision and sensitivity, the preliminary search hedges identified 37.7% (90/239) of articles from MEDLINE and 37.5% (72/192) of articles from APA PsycInfo that met the eligibility criteria, achieving a precision of 39% for both databases. The hedges retrieved all articles from the gold-standard set, achieving a 100% sensitivity rate. Additionally, 5.7% (11/192) of the records from APA PsycInfo were not available in full text.

#### Stage 2: Finalization of the Search Hedges

On the basis of the precision and sensitivity of the preliminary search hedges, the team refined the search strategies to reduce the number of nonrelevant results. On June 7, 2024, we conducted 2 searches using the finalized search hedges, which produced 31,055 references from MEDLINE and 22,924 references from APA PsycInfo (Multimedia Appendix 3).

#### Sample Size Calculations

Informed by the preliminary results of our pilot hedges, we assume that a similar proportion of sampled records will meet the inclusion criteria (approximately 39%) and expect approximately 10% of records to be unretrievable (although our pilot search in APA PsycInfo only showed 6% of records to be unretrievable). For the MEDLINE hedge, a sample size of 2330 will achieve a targeted sensitivity of 100% (99% CI 99.4%-100%). For the APA PsycInfo hedge, a sample size of 2293 will achieve a targeted sensitivity of 100% (99% CI 99.3%-100%).

The calculation of precision for each search hedge requires the specificity rate. Specificity is defined as the proportion of nonrelevant articles correctly not retrieved by a search hedge [21]. In the context of this project, to calculate specificity, all articles indexed in MEDLINE and APA PsycInfo would need to be screened. This is not a feasible option. Thus, specificity will not be calculated. However, we opted for a targeted specificity of 80% (99% CI 77.2%-83%) for MEDLINE and a targeted specificity of 80% (99% CI 77.1%-83%) for APA PsycInfo. These targeted sensitivity and specificity levels correspond to a precision of 76% for both hedges.

### Search Hedge Validation

#### Title and Abstract Eligibility Criteria

The goal of the title and abstract screening process is to identify references with TGD-relevant content. When screening, team members will select 1 of 5 categories for each reference: *Exclude*, *No_Abstrac*t, *Mixed_Topics*, *LGB_Maybe_T*, and *Include*.

**Figure 1:**
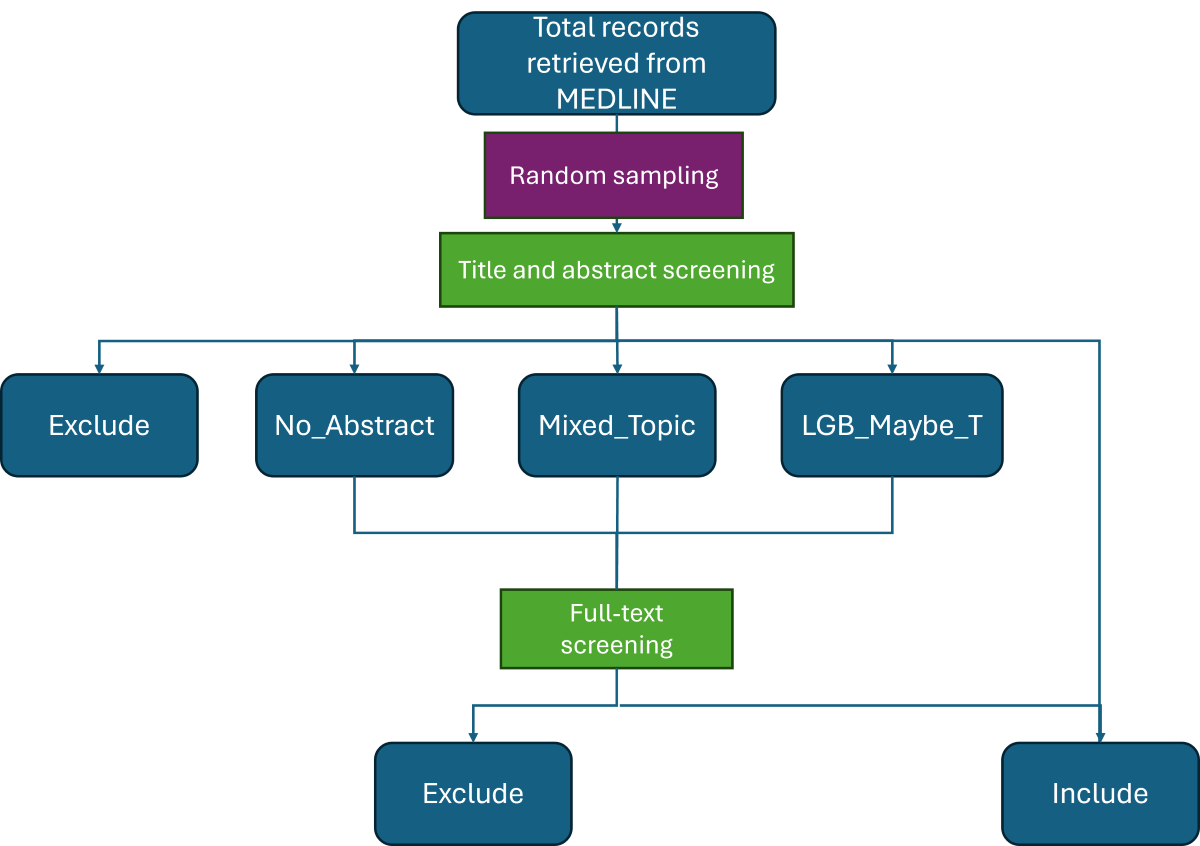
Flow chart depicting the screening process for references relevant to trans and gender diverse (TGD) subject topics from MEDLINE. A similar process was used for results retrieved from APA PsycInfo. Only references categorized as *No_Abstract*, *Mixed_Topics*, or *LGB_Maybe_T* will proceed to the full-text screening phase to validate their relevance to TGD subject topics.

Articles categorized as *Include* or *Exclude* in the screening based on title and abstract phase will not undergo full-text review because each category already demonstrates sufficient data on the relevance or nonrelevance to TGD topic areas, respectively. Abstracts categorized as *No_Abstract*, *Mixed_Topics*, or *LGB_Maybe_T* have insufficient information to determine their relevance and, therefore, will proceed to the full-text screening phase (Figure 1). We will incorporate rates at which references are labeled as *Mixed_Topics* and *LGB_Maybe_T* at the title and abstract screening phase and the rates at which those references with these specific labels are ultimately included after full-text review. Tracking these rates will potentially enable us to derive further semantic clarity on search terms within the hedges, as well as provide recommendations on more targeted use of terminology in future peer-reviewed literature.

#### Exclude Category

References will be excluded if they (1) use solely binary terminology to describe gender (eg, boys and girls, men and women, *both genders*, or *opposite gender*) or (2) focus on psychometric measurement of gender without reporting participants’ self-identification or narrative description (eg, Bem Sex-Role Inventory, including sex typing using feminine, masculine, and androgynous categories) [30]. Abstracts focusing on intersex or DSD topics will also be excluded.

#### Include Category

References will be included if they (1) report on TGD people, or both, in their sample; or (2) specifically discuss TGD communities or TGD topics, such as gender-affirming health care, gender expression, or drag performance. References containing historical terminology referring to Two-Spirit or TGD people, as well as Indigenous and non-Eurocentric gender-expansive terminology, will be included.

#### No_Abstract Category

References without abstracts will be categorized as *No_Abstract* unless their titles clearly indicate relevancy to TGD populations (eg, using the term *transgender* in the title), in which case they will be categorized as *Include*.

#### LGB_Maybe_T or Mixed_Topics Category

For abstracts with unclear subject matter relevant to TGD topic areas, 2 coding options will be available. Abstracts with an unclear TGD or 2SLGBTQIA+ population or TGD or 2SLGBTQIA+–related study topic will be categorized as *Mixed_Topics* (eg, using the term *gender inclusivity* but with no further clarification of whether this means inclusion of TGD people or only cisgender women). If abstracts use the term *transgender* or *trans* solely associated with or as part of elaborating on sexual and gender identity acronyms (eg, *LGBTQ+* or *2SLGBTQIA+*), these references will be categorized as *LGB_Maybe_T.* Abstracts in the *Mixed_Topics* category will be presumed to relate to the cisgender population with unclear relevance to TGD people and topics until further confirmation via the full text. Abstracts in the *LGB_Maybe_T* category will be presumed to relate to the 2SLGBTQIA+ population with unclear relevance to TGD people and topics until further confirmation via the full text.

#### Full-Text Eligibility Criteria

During full-text screening, articles will be categorized as *Include* or *Exclude*. Similar to the screening based on title and abstract phase, the objective is to accurately identify whether the article is relevant to TGD subject matter.

Articles that are included will (1) clearly distinguish among sex, sexual identity, and gender identity; (2) communicate consideration for participants’ gender self-identification and experiences (eg, self-declaration options in a demographic survey); or (3) consider the TGD population as a distinct subpopulation within the 2SLGBTQIA+ umbrella (eg, report on transgender or gender diverse participants separately in a demographic section or as part of the data analysis strategy). For primary research papers, these considerations should be reflected in the Methods or Results sections.

#### Information Sources

No restrictions will be placed on the type of reference, publication date, or language. All efforts will be made to translate articles that are not in English, French, Spanish, or Mandarin using Google Translate or DeepL Translator (DeepL SE), an online translation software [31]. Additional professional translation services will also be sought for articles in languages that are not stated above.

### Study Records

#### Data Management

Results from the MEDLINE and APA PsycInfo searches will be imported to the EPPI-Reviewer online systematic review software [29]. Deduplication will not be conducted as validation and data analysis will be performed separately for each database. Random samples will be allocated using the *Create reference groups* feature in EPPI-Reviewer. Full-text references will be manually or automatically retrieved via Zotero (Corporation for Digital Scholarship), a citation manager, and uploaded to EPPI-Reviewer. EPPI-Reviewer will also be used to track and manage full text retrieval.

#### Selection Process

The review team includes a total of 6 members with lived experience in the TGD community. To ensure intercoder agreement, team member training will consist of a prescreening walkthrough of the eligibility criteria followed by trial coding until the disagreement rate between all coders is equal to or below the recommended 20% [32]. An existing internally circulated team notebook will be created at the start of the screening process to record notable discussions and decisions throughout the process. This notebook will be available to all team members to ensure consistency between coders. Two team members will independently screen each randomly sampled title, abstract, and full text based on the predefined eligibility criteria. Any disagreements will be addressed through discussion facilitated by a third team member, with at least one of the original screening coders present to ensure clarity and context. If differences cannot be resolved through discussion, the project team leads will make the final decision.

### Data Analysis

#### Intercoder Agreement Calculation

The intercoder agreement in each screening phase will be calculated using the following formula [32]:



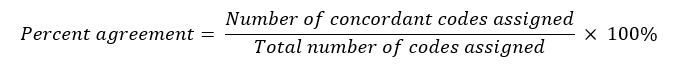



#### Data Items and Planned Statistical Tests

Precision and sensitivity will be calculated to evaluate the validity of each search hedge. Precision quantifies the ability of a search hedge to identify relevant TGD articles. This measurement will be calculated as the number of relevant articles retrieved divided by the total number of screened articles [21]:



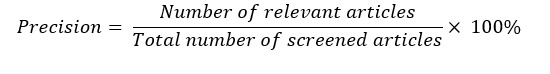



Sensitivity assesses the ability of a search hedge to comprehensively retrieve all relevant articles [21]. Sensitivity will be calculated as the proportion of articles successfully retrieved by the search hedge compared to the gold-standard set as predefined from Knowsy:



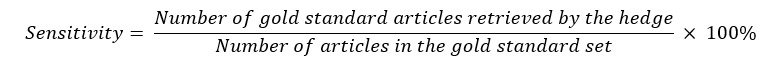



#### Search Term Insights

Polysemic terms identified from the finalized search with unclear relevancy to TGD topics will be assessed. A verbatim search of each term will be conducted in EPPI-Reviewer, and accuracy will be calculated as a proportion of the articles in each category (*Mixed_Topics*, *LGB_Maybe_T*, *Exclude,* and *Include* from both the title and abstract and full-text screening) over the total number of articles containing said term. A higher proportion from the *Mixed_Topics* or *LGB_Maybe_T* category suggests that a term is not being used in a TGD-specific context. A higher proportion from the *Exclude* category suggests that a term does not have high relevance to TGD topics:



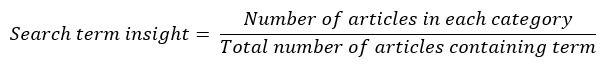



## Results

As of November 2025, we have finished screening all 2330 articles from MEDLINE and all 2293 articles from APA PsycInfo. Data analysis is projected to be finished by December 2025. The results are expected to be published in 2026.

## Discussion

### Expected Findings

While a number of validated and unvalidated search hedges focused on 2SLGBTQIA+ populations exist, to the best of our knowledge, this protocol is the first to detail the process of evaluating 2 search hedges that exclusively focus on the TGD population for MEDLINE and APA PsycInfo [8,9,33,34]. In this paper, we developed a novel screening structure for terminologies with dynamic meanings, with full-text screening only for references where further confirmation was required. We anticipate that both the MEDLINE and APA PsycInfo search hedges will achieve a similar sensitivity of 100% to that of their pilot hedges. We also anticipate that the hedges will achieve higher precision than their pilot counterparts due to the fine-tuning conducted in between. The precision, sensitivity, and search term insight results will provide guidance for librarians and researchers to customize the hedges to best fit their research questions.

While various published and validated search filters exist, most of these hedges focus on retrieving articles based on geographic locations, type of study, and treatment [5,26,27,35]. Validated search hedges focusing on populations, specifically marginalized communities, are particularly scant. Schilperoort et al [34] documented the development and validation process of search filters for PubMed focusing on queer women. Wafford et al [36] documented the development and validation process of a search filter for PubMed focusing on immigrant populations. Both of these population filters relied on the relative recall method to create their respective gold-standard sets. Relative recall is the process of retrieving structured reviews, such as systematic reviews, scoping reviews, and evidence gap maps, related to the specified population and searching through the citations of each review to further identify relevant articles. Schilperoort et al [34] used an internal validation approach in which the gold-standard set was used to generate the search terms as well as the standard for testing the sensitivity of the filters. On the other hand, Wafford et al [36] used an external validation approach in which the team followed the same process of relative recall but partitioned the retrieved articles into 2 separate sets: a development set from which to derive search terms and a validation set (occupying the same role of a gold standard) to test the filter. External validation offers more rigorous results as it mimics a testing environment closer to how a filter will be deployed in a real-world setting [35]. In this protocol, we are able to use an external validation approach due to the existence of the TGD subset in Knowsy. The Knowsy dataset is entirely independent in development and screening to the 2 current search hedges, allowing for a more stringent validation process.

Our validation method further diverged from the methods used by Schilperoort et al [34] and Wafford et al [36]. Both groups validated the precision of their filters against known sets established through the relative recall process, whereas the existence of the Knowsy dataset as our gold standard allows us to test and calculate the precisions and sensitivities of our hedges against unknown sets, offering a closer resemblance to real-world settings [34,36]. While Wafford et al [36] also used external validation, the team only accessed articles at the title and abstract level. For a large portion of our retrieved articles, the titles and abstracts do not provide sufficient information regarding our population of interest, a similar problem that Schilperoort et al [34] also encountered with a search filter focused on queer women. Thus, without the need to generate a gold-standard or validation set, our screening process can explore the semantics and context of terms through our *Mixed_Topics* and *LGB_Maybe_T* categories while minimizing the number of articles needing to be screened in full text.

### Knowledge Mobilization Plan

Once validated, the search hedges will be submitted to the InterTASC Information Specialists’ Sub-Group Search Filter Resource, the McMaster Health Knowledge Refinery, and the University of Alberta Library’s health sciences search filter database [37-39]. We plan to periodically update the 2 hedges to keep abreast with indexing changes in their respective databases. Additionally, future plans will include development and adaptation of the hedges for other bibliographic databases, such as Embase, PubMed, CINAHL, and Scopus. Other knowledge mobilization plans include presentations at relevant research and knowledge synthesis conferences such as Moving Trans History Forward and the Global Evidence Summit [40,41].

### Limitations and Considerations

One primary limitation of the hedges is the limited semantic search. This is related to the standard search mechanisms in MEDLINE, APA PsycInfo, and other databases. The default search mechanism for these databases is the keyword search, which relies on exact match or known variants of the search strings [42]. For example, if a search string specifies the term *transgender*, the keyword search will retrieve only items with the exact match. Truncation operators can increase recall by looking for variants based on the word root (eg, plurals) but not semantic meaning. However, a keyword search does not take into account the context in which the term occurred, thus retrieving results with false semantic matches. In this example, articles with phrases such as *not transgender* would still be retrieved by the hedges. We anticipate that this search mechanism will decrease the precision rates of the hedges.

Another consideration when using the search hedges is the trade-off between sensitivity and precision. The primary user audience for the search hedges are information professionals supporting evidence syntheses, and thus, the hedges were designed with sensitivity as the top priority. The trade-off of this prioritization is precision, and certain types of reviews with time and resource constraints, such as rapid reviews, should keep this in mind. By examining specific search terms that are anticipated to retrieve more nonrelevant articles, we hope to provide librarians and other readers with additional information to make guided adaptations of the hedges that best fit individual research questions and purposes.

### Conclusions

This protocol provides a blueprint for future studies to respectfully and comprehensively search literature related to self-described identities used by TGD communities. This protocol, along with other published articles of its kind, will contribute to the openness of science and the practice of sharing resources within the research community.

## Appendix

Multimedia Appendix 1 Lists of polysemic terms identified during pilot review.

Multimedia Appendix 2 List of trans and gender diverse gold standard references from Knowsy (searched in 2019).

Multimedia Appendix 3 Trans search hedges in Ovid MEDLINE and APA PsycInfo.

## Abbreviations

DSD: difference of sex development
2SLGBTQIA+: Two-Spirit, lesbian, gay, bisexual, transgender, queer, intersex, asexual, and additional sexual and gender diverse terminologies
TGD: transgender and gender diverse

## Data Availability

The datasets generated or analyzed during this study are available from the corresponding author on reasonable request.
