# Supplementary material for: Retrieving Transgender and Gender Diverse Literature: Protocol for the Development and Validation of 2 Search Hedges
Source: JMIR Res Protoc. 2026 Jan 23;15:e76055. doi: 10.2196/76055 (PMC12881892; doi:10.2196/76055)
Supplement: Multimedia Appendix 1 [file resprot_v15i1e76055_app1.docx]

Supplementary Material 1: Lists of polysemic terms identified during pilot review

1. Gender inclusive
2. Gender inclusivity
3. Gender diverse
4. Gender diversity
5. Gender non-conform
6. Gender non-conforming
7. Gender non-conformity
8. Trans person(s)
