# Supplementary material for: Retrieving Transgender and Gender Diverse Literature: Protocol for the Development and Validation of 2 Search Hedges
Source: JMIR Res Protoc. 2026 Jan 23;15:e76055. doi: 10.2196/76055 (PMC12881892; doi:10.2196/76055)
Supplement: Multimedia Appendix 2 [file resprot_v15i1e76055_app2.docx]

Supplementary Material 2: List of Trans and Gender Diverse Gold Standard References from Knowsy 2019

1. Baral SD, Poteat T, Stromdahl S, Wirtz AL, Guadamuz TE, Beyrer C. Worldwide burden of HIV in transgender women: a systematic review and meta-analysis. *The Lancet Infectious diseases*. 2013;13(3):214-222. doi:[10.1016/S1473-3099(12)70315-8](https://doi.org/10.1016/S1473-3099(12)70315-8)

2. Finkenauer S, Sherratt J, Marlow J, Brodey A. When injustice gets old: A systematic review of trans aging. *Journal of Gay & Lesbian Social Services: The Quarterly Journal of Community & Clinical Practice*. 2012;24(4):311-330. doi:[10.1080/10538720.2012.722497](https://doi.org/10.1080/10538720.2012.722497)

3. Morrison Melanie A, Bishop CJ, Morrison Todd G. What is the best measure of discrimination against trans people?: A systematic review of the psychometric literature. *Psychology & Sexuality*. 2018;9(3):269-287. doi:[10.1080/19419899.2018.1484798](https://doi.org/10.1080/19419899.2018.1484798)

4. Davy Z, Toze M. What Is Gender Dysphoria? A Critical Systematic Narrative Review. *Transgender health*. 2018;3(1):159-169. doi:[10.1089/trgh.2018.0014](https://doi.org/10.1089/trgh.2018.0014)

5. Hafford-Letchfield T, Cocker C, Rutter D, Tinarwo M, McCormack K, Manning R. What do we know about transgender parenting?: Findings from a systematic review. *Health & social care in the community*. 2019;27(5):1111-1125. doi:[10.1111/hsc.12759](https://doi.org/10.1111/hsc.12759)

6. McCann E, Brown M. Vulnerability and psychosocial risk factors regarding people who identify as transgender A systematic review of the research evidence. *Issues in Mental Health Nursing*. 2018;39(1):3-15. doi:[10.1080/01612840.2017.1382623](https://doi.org/10.1080/01612840.2017.1382623)

7. Khan J, Schmidt RL, Spittal MJ, Goldstein Z, Smock KJ, Greene DN. Venous Thrombotic Risk in Transgender Women Undergoing Estrogen Therapy: A Systematic Review and Metaanalysis. *Clinical chemistry*. 2019;65(1):57-66. doi:[10.1373/clinchem.2018.288316](https://doi.org/10.1373/clinchem.2018.288316)

8. Adams N, Hitomi M, Moody C. Varied reports of adult transgender suicidality: Synthesizing and describing the peer-reviewed and gray literature. *Transgender Health*. 2017;2(1):60-75. doi:[10.1089/trgh.2016.0036](https://doi.org/10.1089/trgh.2016.0036)

9. Radix A, Sevelius J, Deutsch MB. Transgender women, hormonal therapy and HIV treatment: a comprehensive review of the literature and recommendations for best practices. *Journal of the International AIDS Society*. 2016;19(3 Suppl 2):20810. doi:[10.7448/IAS.19.3.20810](https://doi.org/10.7448/IAS.19.3.20810)

10. Meerwijk E L, Sevelius J M. Transgender Population Size in the United States: a Meta-Regression of Population-Based Probability Samples. *American journal of public health*. 2017;107(2):e1-e8. doi:[10.2105/AJPH.2016.303578](https://doi.org/10.2105/AJPH.2016.303578)

11. Song T E, Jiang N. Transgender Phonosurgery: A Systematic Review and Meta-analysis. *Otolaryngology - Head and Neck Surgery (United States)*. 2017;156(5):803-808. doi:[10.1177/0194599817697050](https://doi.org/10.1177/0194599817697050)

12. Canoy N, Thapa S, Hannes K. Transgender persons’ HIV care (dis)engagement: A qualitative evidence synthesis protocol from an ecological systems theory perspective. *BMJ Open*. 2019;9(1). doi:[10.1136/bmjopen-2018-025475](https://doi.org/10.1136/bmjopen-2018-025475)

13. Heng A, Heal C, Banks J, Preston R. Transgender peoples’ experiences and perspectives about general healthcare: A systematic review. *International Journal of Transgenderism*. 2018;19(4):359-378. doi:[10.1080/15532739.2018.1502711](https://doi.org/10.1080/15532739.2018.1502711)

14. Dubin SN, Nolan IT, Streed CG Jr, Greene RE, Radix AE, Morrison SD. Transgender health care: improving medical students’ and residents’ training and awareness. *Advances in medical education and practice*. 2018;9:377-391. doi:[10.2147/AMEP.S147183](https://doi.org/10.2147/AMEP.S147183)

15. Dowers E, White C, Kingsley J, Swenson R. Transgender experiences of occupation and the environment: A scoping review. *Journal of Occupational Science*. 2019;26(4):496-510. doi:[10.1080/14427591.2018.1561382](https://doi.org/10.1080/14427591.2018.1561382)

16. Perez-Brumer A G, Oldenburg C E, Reisner S L, Clark J L, Parker R G. Towards ‘reflexive epidemiology’: Conflation of cisgender male and transgender women sex workers and implications for global understandings of HIV prevalence. *Global Public Health*. 2016;11(7-8):849-865. doi:[10.1080/17441692.2016.1181193](https://doi.org/10.1080/17441692.2016.1181193)

17. Thorne N, Yip Andrew KT, Bouman Walter P, Marshall E, Arcelus J. The terminology of identities between, outside and beyond the gender binary – A systematic review. *International Journal of Transgenderism*. 2019;20(2/3):138-154. doi:[10.1080/15532739.2019.1640654](https://doi.org/10.1080/15532739.2019.1640654)

18. Stephenson R, Riley E, Rogers E, et al. The Sexual Health of Transgender Men: A Scoping Review. *Journal of sex research*. 2017;54(4-5):424-445. doi:[10.1080/00224499.2016.1271863](https://doi.org/10.1080/00224499.2016.1271863)

19. Nolan IT, Morrison SD, Arowojolu O, et al. The Role of Voice Therapy and Phonosurgery in Transgender Vocal Feminization. *The Journal of craniofacial surgery*. 2019;30(5):1368-1375. doi:[10.1097/SCS.0000000000005132](https://doi.org/10.1097/SCS.0000000000005132)

20. Rooker SA, Vyas KS, DiFilippo EC, Nolan IT, Morrison SD, Santucci RA. The Rise of the Neophallus: A Systematic Review of Penile Prosthetic Outcomes and Complications in Gender-Affirming Surgery. *The journal of sexual medicine*. 2019;16(5):661-672. doi:[10.1016/j.jsxm.2019.03.009](https://doi.org/10.1016/j.jsxm.2019.03.009)

21. Connolly M D, Zervos M J, Barone C J, Johnson C C, Joseph C L.M. The Mental Health of Transgender Youth: Advances in Understanding. *Journal of Adolescent Health*. 2016;59(5):489-495. doi:[10.1016/j.jadohealth.2016.06.012](https://doi.org/10.1016/j.jadohealth.2016.06.012)

22. Restar Arjee J, Surace Anthony, Ogunbajo Adedotun, Edeza Alberto, Kahler Christopher. The HIV-Related Risk Factors of the Cisgender Male Sexual Partners of Transgender Women (MSTW) in the United States: A Systematic Review of the Literature. *AIDS Education & Prevention*. 2019;31(5):463-478. doi:[10.1521/aeap.2019.31.5.463](https://doi.org/10.1521/aeap.2019.31.5.463)

23. Goodrum Benjamin A. The Effects of Long-Term Testosterone Use on Lipid-Related Cardiovascular Risk Factors Among FtM Patients. *International Journal of Transgenderism*. 2012;15(3/4):164-172. doi:[10.1080/15532739.2014.995261](https://doi.org/10.1080/15532739.2014.995261)

24. Rowniak S, Bolt L. The effects of cross-gender hormones on the quality of life of transgender individuals: a systematic review protocol. *JBI database of systematic reviews and implementation reports*. 2015;13(7):93-103. doi:[10.11124/jbisrir-2015-2228](https://doi.org/10.11124/jbisrir-2015-2228)

25. Van Damme S, Cosyns M, Deman S, Van den Eede Z, Van Borsel J. The Effectiveness of Pitch-raising Surgery in Male-to-Female Transsexuals: A Systematic Review. *Journal of Voice*. 2017;31(2):244.e1-244.e5. doi:[10.1016/j.jvoice.2016.04.002](https://doi.org/10.1016/j.jvoice.2016.04.002)

26. Costa Rosalia, Colizzi Marco. The effect of cross-sex hormonal treatment on gender dysphoria individuals’ mental health: A systematic review. *Neuropsychiatric Disease and Treatment*. 2016;4(121953-66. doi: 10.2147/NDT.S95310. eCollection 2016.

27. Fernandez R, Cortes-Cortes J, Esteva I, et al. The CYP17 MspA1 Polymorphism and the Gender Dysphoria. *The journal of sexual medicine*. 2015;12(6):1329-1333. doi:[10.1111/jsm.12895](https://doi.org/10.1111/jsm.12895)

28. Morrison Melanie A, Bishop CJ, Gazzola Stephanie B, McCutcheon Jessica M, Parker Kandice, Morrison Todd G. Systematic review of the psychometric properties of transphobia scales. *International Journal of Transgenderism*. 2017;18(4):395-410. doi:[10.1080/15532739.2017.1332535](https://doi.org/10.1080/15532739.2017.1332535)

29. Delgado-Ruiz R, Swanson P, Romanos G. Systematic review of the long-term effects of transgender hormone therapy on bone markers and bone mineral density and their potential effects in implant therapy. *Journal of Clinical Medicine*. 2019;8(6). doi:[10.3390/jcm8060784](https://doi.org/10.3390/jcm8060784)

30. Cocci A, Frediani D, Cacciamani GE, et al. Systematic review of studies reporting perioperative and functional outcomes following male-to- female gender assignment surgery (MtoF GAS): a call for standardization in data reporting. *Minerva urologica e nefrologica = The Italian journal of urology and nephrology*. Published online May 2019. doi:[10.23736/S0393-2249.19.03407-6](https://doi.org/10.23736/S0393-2249.19.03407-6)

31. Karamitros GA, Kitsos NA, Sapountzis S. Systematic Review of Quality of Patient Information on Phalloplasty in the Internet. *Aesthetic plastic surgery*. 2017;41(6):1426-1434. doi:[10.1007/s00266-017-0937-5](https://doi.org/10.1007/s00266-017-0937-5)

32. Arcelus J, Bouman W P, Van Den Noortgate W, Claes L, Witcomb G, Fernandez-Aranda F. Systematic review and meta-analysis of prevalence studies in transsexualism. *European Psychiatry*. 2015;30(6):807-815. doi:[10.1016/j.eurpsy.2015.04.005](https://doi.org/10.1016/j.eurpsy.2015.04.005)

33. van der Sluis WB, Buncamper ME, Bouman MB, et al. Symptomatic HPV-related neovaginal lesions in transgender women: case series and review of literature. *Sexually transmitted infections*. 2016;92(7):499-501. doi:[10.1136/sextrans-2015-052456](https://doi.org/10.1136/sextrans-2015-052456)

34. McNeil Jay, Ellis Sonja J, Eccles Fiona J. R. Suicide in trans populations: A systematic review of prevalence and correlates. *Psychology of Sexual Orientation and Gender Diversity*. 2017;4(3):341-353. doi:[10.1037/sgd0000235](https://doi.org/10.1037/sgd0000235)

35. Magno L, Silva LAVD, Veras MA, Pereira-Santos M, Dourado I. Stigma and discrimination related to gender identity and vulnerability to HIV/AIDS among transgender women: a systematic review. *Cadernos de saude publica*. 2019;35(4):e00112718. doi:[10.1590/0102-311X00112718](https://doi.org/10.1590/0102-311X00112718)

36. Jones Bethany Alice, Arcelus Jon, Bouman Walter Pierre, Haycraft Emma. Sport and transgender people: A systematic review of the literature relating to sport participation and competitive sport policies. *Sports Medicine*. 2017;47(4):701-716. doi:[10.1007/s40279-016-0621-y](https://doi.org/10.1007/s40279-016-0621-y)

37. Wei C, Herrick A, Raymond HF, Anglemyer A, Gerbase A, Noar SM. Social marketing interventions to increase HIV/STI testing uptake among men who have sex with men and male-to-female transgender women. *Cochrane Database of Systematic Reviews*. Published online January 2011:N.PAG-N.PAG.

38. Nieder T O, Elaut E, Richards C, Dekker A. Sexual orientation of trans adults is not linked to outcome of transition-related health care, but worth asking. *International Review of Psychiatry*. 2016;28(1):103-111. doi:[10.3109/09540261.2015.1102127](https://doi.org/10.3109/09540261.2015.1102127)

39. Operario D, Soma T, Underhill K. Sex work and HIV status among transgender women: systematic review and meta-analysis. *Journal of acquired immune deficiency syndromes (1999)*. 2008;48(1):97-103. doi:[10.1097/QAI.0b013e31816e3971](https://doi.org/10.1097/QAI.0b013e31816e3971)

40. Maraka S, Ospina N S, Rodriguez-Gutierrez R, et al. Sex steroids and cardiovascular outcomes in transgender individuals: A systematic review and meta-analysis. *Journal of Clinical Endocrinology and Metabolism*. 2017;102(11):3914-3923. doi:[10.1210/jc.2017-01643](https://doi.org/10.1210/jc.2017-01643)

41. Kennedy CE, Bernard LJ, Muessig KE, et al. Serosorting and HIV/STI Infection among HIV-Negative MSM and Transgender People: A Systematic Review and Meta-Analysis to Inform WHO Guidelines. *Journal of sexually transmitted diseases*. 2013;2013:583627. doi:[10.1155/2013/583627](https://doi.org/10.1155/2013/583627)

42. Wanta JW, Unger CA. Review of the Transgender Literature: Where Do We Go from Here? *Transgender health*. 2017;2(1):119-128. doi:[10.1089/trgh.2017.0004](https://doi.org/10.1089/trgh.2017.0004)

43. Wylie KR, Fung R Jr, Boshier C, Rotchell M. Recommendations of endocrine treatment for patients with gender dysphoria. *Sexual & Relationship Therapy*. 2009;24(2):175-187.

44. Nobili A, Glazebrook C, Arcelus J. Quality of life of treatment-seeking transgender adults: A systematic review and meta-analysis. *Reviews in Endocrine and Metabolic Disorders*. 2018;19(3):199-220. doi:[10.1007/s11154-018-9459-y](https://doi.org/10.1007/s11154-018-9459-y)

45. Weinforth G, Fakin R, Giovanoli P, Nunez DG. Quality of Life Following Male-To-Female Sex Reassignment Surgery. *Deutsches Arzteblatt international*. 2019;116(15):253-260. doi:[10.3238/arztebl.2019.0253](https://doi.org/10.3238/arztebl.2019.0253)

46. Passos Taciana Silveira, Teixeira Marina Sá, Almeida-Santos Marcos Antonio. Quality of life after gender affirmation surgery: A systematic review and network meta-analysis. *Sexuality Research & Social Policy: A Journal of the NSRC*. Published online June 2019. doi:[10.1007/s13178-019-00394-0](https://doi.org/10.1007/s13178-019-00394-0)

47. Catelan Ramiro Figueiredo, Costa Angelo Brandelli, Lisboa Carolina Saraiva de Macedo. Psychological Interventions for Transgender Persons: A Scoping Review. *International Journal of Sexual Health*. 2017;29(4):325-337. doi:[10.1080/19317611.2017.1360432](https://doi.org/10.1080/19317611.2017.1360432)

48. Johns M M, Beltran O, Armstrong H L, Jayne P E, Barrios L C. Protective Factors Among Transgender and Gender Variant Youth: A Systematic Review by Socioecological Level. *The journal of primary prevention*. 2018;39(3):263-301. doi:[10.1007/s10935-018-0508-9](https://doi.org/10.1007/s10935-018-0508-9)

49. Kcomt Luisa. Profound health-care discrimination experienced by transgender people: rapid systematic review. *Social Work in Health Care*. 2019;58(2):201-219. doi:[10.1080/00981389.2018.1532941](https://doi.org/10.1080/00981389.2018.1532941)

50. Collin L, Reisner SL, Tangpricha V, Goodman M. Prevalence of Transgender Depends on the “Case” Definition: A Systematic Review. *The journal of sexual medicine*. 2016;13(4):613-626. doi:[10.1016/j.jsxm.2016.02.001](https://doi.org/10.1016/j.jsxm.2016.02.001)

51. Millet Nessa, Longworth Julia, Arcelus Jon. Prevalence of anxiety symptoms and disorders in the transgender population: A systematic review of the literature. *International Journal of Transgenderism*. 2017;18(1):27-38. doi:[10.1080/15532739.2016.1258353](https://doi.org/10.1080/15532739.2016.1258353)

52. D’Andrea S, Pallotti F, Senofonte G, et al. Polymorphic Cytosine-Adenine-Guanine Repeat Length of Androgen Receptor Gene and Gender Incongruence in Trans Women: A Systematic Review and Meta-Analysis of Case-Control Studies. *Journal of Sexual Medicine*. 2020;17(3):543-550. doi:[10.1016/j.jsxm.2019.12.010](https://doi.org/10.1016/j.jsxm.2019.12.010)

53. Morrison SD, Shakir A, Vyas KS, Kirby J, Crane CN, Lee GK. Phalloplasty: A Review of Techniques and Outcomes. *Plastic and reconstructive surgery*. 2016;138(3):594-615. doi:[10.1097/PRS.0000000000002518](https://doi.org/10.1097/PRS.0000000000002518)

54. Andreasson M, Georgas K, Elander A, Selvaggi G. Patient-Reported Outcome Measures Used in Gender Confirmation Surgery: A Systematic Review. *Plastic and reconstructive surgery*. 2018;141(4):1026-1039. doi:[10.1097/PRS.0000000000004254](https://doi.org/10.1097/PRS.0000000000004254)

55. Remington AC, Morrison SD, Massie JP, et al. Outcomes after Phalloplasty: Do Transgender Patients and Multiple Urethral Procedures Carry a Higher Rate of Complication? *Plastic and reconstructive surgery*. 2018;141(2):220e-229e. doi:[10.1097/PRS.0000000000004061](https://doi.org/10.1097/PRS.0000000000004061)

56. Horbach S E.R, Bouman M B, Smit J M, Özer M, Buncamper M E, Mullender M G. Outcome of Vaginoplasty in Male-to-Female Transgenders: A Systematic Review of Surgical Techniques. *Journal of Sexual Medicine*. 2015;12(6):1499-1512. doi:[10.1111/jsm.12868](https://doi.org/10.1111/jsm.12868)

57. Edmiston E K, Donald C A, Sattler A R, Peebles J K, Ehrenfeld J M, Eckstrand K L. Opportunities and Gaps in Primary Care Preventative Health Services for Transgender Patients: A Systematic Review. *Transgender Health*. 2016;1(1):216-230. doi:[10.1089/trgh.2016.0019](https://doi.org/10.1089/trgh.2016.0019)

58. Melendez Rita M, Bonem Lathem A, Sember Robert. On Bodies and Research: Transgender Issues in Health and HIV Research Articles. *Sexuality Research & Social Policy*. 2006;3(4):21-38. doi:[10.1525/srsp.2006.3.4.21](https://doi.org/10.1525/srsp.2006.3.4.21)

59. Rosa DF, Carvalho MVF, Pereira NR, Rocha NT, Neves VR, Rosa ADS. Nursing Care for the transgender population: genders from the perspective of professional practice. *Revista brasileira de enfermagem*. 2019;72(suppl 1):299-306. doi:[10.1590/0034-7167-2017-0644](https://doi.org/10.1590/0034-7167-2017-0644)

60. Ascha M, Swanson MA, Massie JP, et al. Nonsurgical Management of Facial Masculinization and Feminization. *Aesthetic surgery journal*. 2019;39(5):NP123-NP137. doi:[10.1093/asj/sjy253](https://doi.org/10.1093/asj/sjy253)

61. Marshall Ellen, Claes Laurence, Bouman Walter Pierre, Witcomb Gemma L, Arcelus Jon. Non-suicidal self-injury and suicidality in trans people: A systematic review of the literature. *International Review of Psychiatry*. 2016;28(1):58-69. doi:[10.3109/09540261.2015.1073143](https://doi.org/10.3109/09540261.2015.1073143)

62. Altinay M, Anand A. Neuroimaging gender dysphoria: a novel psychobiological model. *Brain imaging and behavior*. Published online 2019. doi:[10.1007/s11682-019-00121-8](https://doi.org/10.1007/s11682-019-00121-8)

63. Macri Diana, Wolfe Kate. My preferred pronoun is she: Understanding transgender identity and oral health care needs. *Canadian Journal of Dental Hygiene*. 2019;53(2):110-117.

64. Brown Suzanne, Kucharska Jo, Marczak Magdalena. Mental health practitioners’ attitudes towards transgender people: A systematic review of the literature. *International Journal of Transgenderism*. 2018;19(1):4-24. doi:[10.1080/15532739.2017.1374227](https://doi.org/10.1080/15532739.2017.1374227)

65. McCann E, Sharek D. Mental Health Needs of People Who Identify as Transgender: A Review of the Literature. *Archives of psychiatric nursing*. 2016;30(2):280-285. doi:[10.1016/j.apnu.2015.07.003](https://doi.org/10.1016/j.apnu.2015.07.003)

66. Wilson SC, Morrison SD, Anzai L, et al. Masculinizing Top Surgery: A Systematic Review of Techniques and Outcomes. *Annals of plastic surgery*. 2018;80(6):679-683. doi:[10.1097/SAP.0000000000001354](https://doi.org/10.1097/SAP.0000000000001354)

67. Goldstein Z, Khan M, Reisman T, Safer JD. Managing the risk of venous thromboembolism in transgender adults undergoing hormone therapy. *Journal of blood medicine*. 2019;10:209-216. doi:[10.2147/JBM.S166780](https://doi.org/10.2147/JBM.S166780)

68. Stotzer R L. Law enforcement and criminal justice personnel interactions with transgender people in the United States: A literature review. *Aggression and Violent Behavior*. 2014;19(3):263-277. doi:[10.1016/j.avb.2014.04.012](https://doi.org/10.1016/j.avb.2014.04.012)

69. Schwarz K, Fontanari A M.V, Schneider M A, et al. Laryngeal surgical treatment in transgender women: A systematic review and meta-analysis. *Laryngoscope*. 2017;127(11):2596-2603. doi:[10.1002/lary.26692](https://doi.org/10.1002/lary.26692)

70. Anderson James E, Kanters Steve. Lack of sexual minorities’ rights as a barrier to HIV prevention among men who have sex with men and transgender women in Asia: A systematic review. *LGBT Health*. 2015;2(1):16-26. doi:[10.1089/lgbt.2014.0024](https://doi.org/10.1089/lgbt.2014.0024)

71. Sullivan Patrick, Trinidad John, Hamann Dathan. Issues in transgender dermatology: A systematic review of the literature. *Journal of the American Academy of Dermatology*. 2019;81(2):438-447. doi:[10.1016/j.jaad.2019.03.023](https://doi.org/10.1016/j.jaad.2019.03.023)

72. Stewart Lauryn, O’Halloran Paul, Oates Jennifer. Investigating the social integration and wellbeing of transgender individuals: A meta-synthesis. *International Journal of Transgenderism*. 2018;19(1):46-58. doi:[10.1080/15532739.2017.1364199](https://doi.org/10.1080/15532739.2017.1364199)

73. Newman-Valentine D, Duma S. Injustice to transsexual women in a hetero-normative healthcare system. *African journal of primary health care & family medicine*. 2014;6(1):E1-E5. doi:[10.4102/phcfm.v6i1.574](https://doi.org/10.4102/phcfm.v6i1.574)

74. Phillips Gregory, Peterson James, Binson Diane, Hidalgo Julia, Magnus Manya. House/ball culture and adolescent African-American transgender persons and men who have sex with men: a synthesis of the literature. *AIDS Care*. 2011;23(4):515-520. doi:[10.1080/09540121.2010.516334](https://doi.org/10.1080/09540121.2010.516334)

75. Chew D, Anderson J, Williams K, May T, Pang K. Hormonal treatment in young people with gender dysphoria: A systematic review. *Pediatrics*. 2018;141(4). doi:[10.1542/peds.2017-3742](https://doi.org/10.1542/peds.2017-3742)

76. Murad MH, Elamin MB, Garcia MZ, et al. Hormonal therapy and sex reassignment: a systematic review and meta-analysis of quality of life and psychosocial outcomes. *Clinical endocrinology*. 2010;72(2):214-231. doi:[10.1111/j.1365-2265.2009.03625.x](https://doi.org/10.1111/j.1365-2265.2009.03625.x)

77. Rao A, Schwartz S, Sabin K, et al. HIV-related data among key populations to inform evidence-based responses: protocol of a systematic review. *Systematic reviews*. 2018;7(1):220. doi:[10.1186/s13643-018-0894-3](https://doi.org/10.1186/s13643-018-0894-3)

78. Vaitses Fontanari, Anna Martha, Zanella Gabriel Ibarra, et al. HIV-related care for transgender people: A systematic review of studies from around the world. *Social Science & Medicine*. 2019;230:280-294. doi:[10.1016/j.socscimed.2019.03.016](https://doi.org/10.1016/j.socscimed.2019.03.016)

79. Giami A, Le Bail J. HIV infection and STI in the trans population: A critical review. *Revue d’Epidemiologie et de Sante Publique*. 2011;59(4):259-268. doi:[10.1016/j.respe.2011.02.102](https://doi.org/10.1016/j.respe.2011.02.102)

80. Tang S, Tang W, Meyers K, Chan P, Chen Z, Tucker J D. HIV epidemiology and responses among men who have sex with men and transgender individuals in China: A scoping review. *BMC Infectious Diseases*. 2016;16(1). doi:[10.1186/s12879-016-1904-5](https://doi.org/10.1186/s12879-016-1904-5)

81. Defreyne Justine, Motmans Joz, T’sjoen Guy, T’sjoen Guy. Healthcare costs and quality of life outcomes following gender affirming surgery in trans men: a review. *Expert Review of Pharmacoeconomics & Outcomes Research*. 2017;17(6):543-556. doi:[10.1080/14737167.2017.1388164](https://doi.org/10.1080/14737167.2017.1388164)

82. Scandurra C, Mezza F, Maldonato NM, et al. Health of Non-binary and Genderqueer People: A Systematic Review. *Frontiers in psychology*. 2019;10:1453. doi:[10.3389/fpsyg.2019.01453](https://doi.org/10.3389/fpsyg.2019.01453)

83. Patel Jharna M, Dolitsky Shelley, Bachman Gloria A, Buckley de Meritens, Alexandre. Gynecologic cancer screening in the transgender male population and its current challenges. *Maturitas*. 2019;129:40-44. doi:[10.1016/j.maturitas.2019.08.009](https://doi.org/10.1016/j.maturitas.2019.08.009)

84. Poteat T, Scheim A, Xavier J, Reisner S, Baral S. Global Epidemiology of HIV Infection and Related Syndemics Affecting Transgender People. *Journal of acquired immune deficiency syndromes (1999)*. 2016;72 Suppl 3:S210-9. doi:[10.1097/QAI.0000000000001087](https://doi.org/10.1097/QAI.0000000000001087)

85. Dunford C, Bell K, Rashid T. Genital Reconstructive Surgery in Male to Female Transgender Patients: A Systematic Review of Primary Surgical Techniques, Complication Profiles, and Functional Outcomes from 1950 to Present Day. *European Urology Focus*. Published online 2020:1-8. doi:[10.1016/j.euf.2020.01.004](https://doi.org/10.1016/j.euf.2020.01.004)

86. McFarlane T, Zajac JD, Cheung AS. Gender-affirming hormone therapy and the risk of sex hormone-dependent tumours in transgender individuals-A systematic review. *Clinical endocrinology*. 2018;89(6):700-711. doi:[10.1111/cen.13835](https://doi.org/10.1111/cen.13835)

87. de Jesus LE, Costa EC, Dekermacher S. Gender dysphoria and XX congenital adrenal hyperplasia: how frequent is it? Is male-sex rearing a good idea? *Journal of pediatric surgery*. Published online February 2019. doi:[10.1016/j.jpedsurg.2019.01.062](https://doi.org/10.1016/j.jpedsurg.2019.01.062)

88. Glidden D, Bouman WP, Jones BA, Arcelus J. Gender Dysphoria and Autism Spectrum Disorder: A Systematic Review of the Literature. *Sexual medicine reviews*. 2016;4(1):3-14. doi:[10.1016/j.sxmr.2015.10.003](https://doi.org/10.1016/j.sxmr.2015.10.003)

89. Van Der Miesen A I.R, Hurley H, De Vries A L.C. Gender dysphoria and autism spectrum disorder: A narrative review. *International Review of Psychiatry*. 2016;28(1):70-80. doi:[10.3109/09540261.2015.1111199](https://doi.org/10.3109/09540261.2015.1111199)

90. Wood E, Halder N. Gender disorders in learning disability -- a systematic review. *Tizard Learning Disability Review*. 2014;19(4):1-1.

91. Cohen WA, Shah NR, Iwanicki M, Therattil PJ, Keith JD. Female-to-Male Transgender Chest Contouring: A Systematic Review of Outcomes and Knowledge Gaps. *Annals of plastic surgery*. Published online May 2019. doi:[10.1097/SAP.0000000000001896](https://doi.org/10.1097/SAP.0000000000001896)

92. Sayegh F, Ludwig DC, Ascha M, et al. Facial Masculinization Surgery and its Role in the Treatment of Gender Dysphoria. *The Journal of craniofacial surgery*. 2019;30(5):1339-1346. doi:[10.1097/SCS.0000000000005101](https://doi.org/10.1097/SCS.0000000000005101)

93. Morrison SD, Vyas KS, Motakef S, et al. Facial Feminization: Systematic Review of the Literature. *Plastic and reconstructive surgery*. 2016;137(6):1759-1770. doi:[10.1097/PRS.0000000000002171](https://doi.org/10.1097/PRS.0000000000002171)

94. Brömdal Annette, Mullens Amy B, Phillips Tania M, Gow Jeff. Experiences of transgender prisoners and their knowledge, attitudes, and practices regarding sexual behaviors and hiv/stis: A systematic review. *International Journal of Transgenderism*. Published online November 2018. doi:[10.1080/15532739.2018.1538838](https://doi.org/10.1080/15532739.2018.1538838)

95. Sbragia J D, Vottero B. Experiences of transgender men in seeking gynecological and reproductive health care: A qualitative systematic review protocol. *JBI Database of Systematic Reviews and Implementation Reports*. 2019;17(8):1582-1588. doi:[10.11124/JBISRIR-2017-004029](https://doi.org/10.11124/JBISRIR-2017-004029)

96. Sbragia J D, Vottero B. Experiences of transgender men in seeking gynecological and reproductive health care: a qualitative systematic review. *JBI Evidence Synthesis*. 2020;18(0):1-59. doi:[10.11124/JBISRIR-D-19-00347](https://doi.org/10.11124/JBISRIR-D-19-00347)

97. Valenta Tamera, Shade Kate, Lieggi Michelle. Experiences of transgender individuals when accessing health care: a qualitative systematic review protocol. *JBI Database of Systematic Reviews & Implementation Reports*. 2018;16(3):628-634. doi:[10.11124/JBISRIR-2017-003438](https://doi.org/10.11124/JBISRIR-2017-003438)

98. Sutcliffe P A, Dixon S, Akehurst R L, et al. Evaluation of surgical procedures for sex reassignment: a systematic review. *Journal of Plastic, Reconstructive and Aesthetic Surgery*. 2009;62(3):294-306. doi:[10.1016/j.bjps.2007.12.009](https://doi.org/10.1016/j.bjps.2007.12.009)

99. Becasen Jeffrey S, Denard Christa L, Mullins Mary M, Higa Darrel H, Sipe Theresa Ann. Estimating the prevalence of HIV and sexual behaviors among the US transgender population: A systematic review and meta-analysis, 2006–2017. *American Journal of Public Health*. 2019;109(1):e1-e8. doi:[10.2105/AJPH.2018.304727](https://doi.org/10.2105/AJPH.2018.304727)

100. Herbst JH, Jacobs ED, Finlayson TJ, McKleroy VS, Neumann MS, Crepaz N. Estimating HIV prevalence and risk behaviors of transgender persons in the United States: a systematic review. *AIDS and behavior*. 2008;12(1):1-17. doi:[10.1007/s10461-007-9299-3](https://doi.org/10.1007/s10461-007-9299-3)

101. Velho I, Fighera TM, Ziegelmann PK, Spritzer PM. Effects of testosterone therapy on BMI, blood pressure, and laboratory profile of transgender men: a systematic review. *Andrology*. 2017;5(5):881-888. doi:[10.1111/andr.12382](https://doi.org/10.1111/andr.12382)

102. Defreyne J, Van de Bruaene L L, Rietzschel E, Van Schuylenbergh J, T’Sjoen G G.R. Effects of gender-affirming hormones on lipid, metabolic, and cardiac surrogate blood markers in transgender persons. *Clinical Chemistry*. 2019;65(1):119-134. doi:[10.1373/clinchem.2018.288241](https://doi.org/10.1373/clinchem.2018.288241)

103. Ziegler Aaron, Henke Travis, Wiedrick Jack, Helou Leah B. Effectiveness of testosterone therapy for masculinizing voice in transgender patients: A meta-analytic review. *International Journal of Transgenderism*. 2018;19(1):25-45. doi:[10.1080/15532739.2017.1411857](https://doi.org/10.1080/15532739.2017.1411857)

104. Singh-Ospina N, Maraka S, Rodriguez-Gutierrez R, et al. Effect of sex steroids on the bone health of transgender individuals: A systematic review and meta-analysis. *Journal of Clinical Endocrinology and Metabolism*. 2017;102(11):3904-3913. doi:[10.1210/jc.2017-01642](https://doi.org/10.1210/jc.2017-01642)

105. Elamin MB, Garcia MZ, Murad MH, Erwin PJ, Montori VM. Effect of sex steroid use on cardiovascular risk in transsexual individuals: a systematic review and meta-analyses. *Clinical endocrinology*. 2010;72(1):1-10. doi:[10.1111/j.1365-2265.2009.03632.x](https://doi.org/10.1111/j.1365-2265.2009.03632.x)

106. Rowniak S, Bolt L, Sharifi C. Effect of cross-sex hormones on the quality of life, depression and anxiety of transgender individuals: a quantitative systematic review. *JBI database of systematic reviews and implementation reports*. 2019;17(9):1826-1854. doi:[10.11124/JBISRIR-2017-003869](https://doi.org/10.11124/JBISRIR-2017-003869)

107. Marshall Z, Welch V, Minichiello A, Swab M, Brunger F, Kaposy C. Documenting Research with Transgender, Nonbinary, and Other Gender Diverse (Trans) Individuals and Communities: Introducing the Global Trans Research Evidence Map. *Transgender Health*. 2019;4(1):68-80. doi:[10.1089/trgh.2018.0020](https://doi.org/10.1089/trgh.2018.0020)

108. McCann E, Brown M. Discrimination and resilience and the needs of people who identify as Transgender: A narrative review of quantitative research studies. *Journal of clinical nursing*. 2017;26(23-24):4080-4093. doi:[10.1111/jocn.13913](https://doi.org/10.1111/jocn.13913)

109. Verbruggen C, Weigert R, Corre P, Casoli V, Bondaz M. [Development of the facial feminization surgery patient’s satisfaction questionnaire (QESFF1): Qualitative phase]. *Annales de chirurgie plastique et esthetique*. 2018;63(3):205-214. doi:[10.1016/j.anplas.2017.12.003](https://doi.org/10.1016/j.anplas.2017.12.003)

110. Marks DH, Awosika O, Rengifo-Pardo M, Ehrlich A. Dermatologic Surgical Care for Transgender Individuals. *Dermatologic surgery*. 2019;45(3):446-457. doi:[10.1097/DSS.0000000000001718](https://doi.org/10.1097/DSS.0000000000001718)

111. MacCarthy S, Poteat T, Xia Z, et al. Current research gaps: a global systematic review of HIV and sexually transmissible infections among transgender populations. *Sexual health*. 2017;14(5):456-468. doi:[10.1071/SH17096](https://doi.org/10.1071/SH17096)

112. Klaver M, Dekker M J.H.J, de Mutsert R, Twisk J W.R, den Heijer M. Cross-sex hormone therapy in transgender persons affects total body weight, body fat and lean body mass: a meta-analysis. *Andrologia*. 2017;49(5). doi:[10.1111/and.12660](https://doi.org/10.1111/and.12660)

113. Wolford-Clevenger C, Frantell K, Smith PN, Flores LY, Stuart GL. Correlates of suicide ideation and behaviors among transgender people: A systematic review guided by ideation-to-action theory. *Clinical psychology review*. 2018;63:93-105. doi:[10.1016/j.cpr.2018.06.009](https://doi.org/10.1016/j.cpr.2018.06.009)

114. Wright T, Candy B, King M. Conversion therapies and access to transition-related healthcare in transgender people: A narrative systematic review. *BMJ Open*. 2018;8(12). doi:[10.1136/bmjopen-2018-022425](https://doi.org/10.1136/bmjopen-2018-022425)

115. Dreher P C, Edwards D, Hager S, et al. Complications of the neovagina in male-to-female transgender surgery: A systematic review and meta-analysis with discussion of management. *Clinical Anatomy*. 2018;31(2):191-199. doi:[10.1002/ca.23001](https://doi.org/10.1002/ca.23001)

116. Manrique OJ, Adabi K, Martinez-Jorge J, Ciudad P, Nicoli F, Kiranantawat K. Complications and Patient-Reported Outcomes in Male-to-Female Vaginoplasty-Where We Are Today: A Systematic Review and Meta-Analysis. *Annals of plastic surgery*. 2018;80(6):684-691. doi:[10.1097/SAP.0000000000001393](https://doi.org/10.1097/SAP.0000000000001393)

117. Wibowo E. Cognitive impacts of estrogen treatment in androgen-deprived males: What needs to be resolved. *Current Neuropharmacology*. 2017;15(7):1043-1055. doi:[10.2174/1570159X15666170313122555](https://doi.org/10.2174/1570159X15666170313122555)

118. Wierckx K, Gooren L, T’Sjoen G. Clinical review: Breast development in trans women receiving cross-sex hormones. *The journal of sexual medicine*. 2014;11(5):1240-1247. doi:[10.1111/jsm.12487](https://doi.org/10.1111/jsm.12487)

119. Tobin Valerie, Delaney Kathleen R. Child abuse victimization among transgender and gender nonconforming people: A systematic review. *Perspectives in Psychiatric Care*. 2019;55(4):576-583. doi:[10.1111/ppc.12398](https://doi.org/10.1111/ppc.12398)

120. Wamboldt R, Haseeb S, Waddington A, Baranchuk A. Cardiac arrhythmias secondary to hormone therapy in trans women. *Expert Review of Cardiovascular Therapy*. 2019;17(5):335-343. doi:[10.1080/14779072.2019.1606713](https://doi.org/10.1080/14779072.2019.1606713)

121. Watters Yulia, Harsh Jennifer, Corbett Cheyenne. Cancer care for transgender patients: Systematic literature review. *International Journal of Transgenderism*. 2014;15(3-4):136-145. doi:[10.1080/15532739.2014.960638](https://doi.org/10.1080/15532739.2014.960638)

122. Maycock LB, Kennedy HP. Breast care in the transgender individual. *Journal of midwifery & women’s health*. 2014;59(1):74-81. doi:[10.1111/jmwh.12066](https://doi.org/10.1111/jmwh.12066)

123. Stone J P, Hartley R L, Temple-Oberle C. Breast cancer in transgender patients: A systematic review. Part 2: Female to Male. *European Journal of Surgical Oncology*. 2018;44(10):1463-1468. doi:[10.1016/j.ejso.2018.06.021](https://doi.org/10.1016/j.ejso.2018.06.021)

124. Hartley R L, Stone J P, Temple-Oberle C. Breast cancer in transgender patients: A systematic review. Part 1: Male to female. *European Journal of Surgical Oncology*. 2018;44(10):1455-1462. doi:[10.1016/j.ejso.2018.06.035](https://doi.org/10.1016/j.ejso.2018.06.035)

125. Joint R, Chen ZE, Cameron S. Breast and reproductive cancers in the transgender population: a systematic review. *BJOG : an international journal of obstetrics and gynaecology*. 2018;125(12):1505-1512. doi:[10.1111/1471-0528.15258](https://doi.org/10.1111/1471-0528.15258)

126. Georgas K, Belgrano V, Andreasson M, Elander A, Selvaggi G. Bowel vaginoplasty: a systematic review. *Journal of plastic surgery and hand surgery*. 2018;52(5):265-273. doi:[10.1080/2000656X.2018.1482220](https://doi.org/10.1080/2000656X.2018.1482220)

127. Fighera T M, Ziegelmann P K, Da Silva T R, Spritzer P M. Bone mass effects of cross-sex hormone therapy in transgender people: Updated systematic review and meta-analysis. *Journal of the Endocrine Society*. 2019;3(5):943-964. doi:[10.1210/js.2018-00413](https://doi.org/10.1210/js.2018-00413)

128. Jones Bethany Alice, Haycraft Emma, Murjan Sarah, Arcelus Jon. Body dissatisfaction and disordered eating in trans people: A systematic review of the literature. *International Review of Psychiatry*. 2016;28(1):81-94. doi:[10.3109/09540261.2015.1089217](https://doi.org/10.3109/09540261.2015.1089217)

129. Sweileh W M. Bibliometric analysis of peer-reviewed literature in transgender health (1900 - 2017). *BMC International Health and Human Rights*. 2018;18(1). doi:[10.1186/s12914-018-0155-5](https://doi.org/10.1186/s12914-018-0155-5)

130. Nugroho A, Erasmus V, Zomer TP, Wu Q, Richardus JH. Behavioral interventions to reduce HIV risk behavior for MSM and transwomen in Southeast Asia: a systematic review. *AIDS care*. 2017;29(1):98-104. doi:[10.1080/09540121.2016.1200713](https://doi.org/10.1080/09540121.2016.1200713)

131. Snow Annie, Cerel Julie, Loeffler Diane N, Flaherty Chris. Barriers to Mental Health Care for Transgender and Gender-Nonconforming Adults: A Systematic Literature Review. *Health & Social Work*. 2019;44(3):149-155. doi:[10.1093/hsw/hlz016](https://doi.org/10.1093/hsw/hlz016)

132. Brookfield S, Dean J, Forrest C, Jones J, Fitzgerald L. Barriers to Accessing Sexual Health Services for Transgender and Male Sex Workers: A Systematic Qualitative Meta-summary. *AIDS and behavior*. Published online March 2019. doi:[10.1007/s10461-019-02453-4](https://doi.org/10.1007/s10461-019-02453-4)

133.Aylagas-Crespillo M, Garcia-Barbero O, Rodriguez-Martin B. Barriers in the social and healthcare assistance for transgender persons: A systematic review of qualitative studies. *Enfermeria clinica*. Published online November 2017. doi:[10.1016/j.enfcli.2017.09.004](https://doi.org/10.1016/j.enfcli.2017.09.004)

134. Boskey E R, Taghinia A H, Ganor O. Association of Surgical Risk with Exogenous Hormone Use in Transgender Patients: A Systematic Review. *JAMA Surgery*. 2019;154(2):109-115. doi:[10.1001/jamasurg.2018.4598](https://doi.org/10.1001/jamasurg.2018.4598)

135. Smith JR, Washington AZ 3rd, Morrison SD, Gottlieb LJ. Assessing Patient Satisfaction Among Transgender Individuals Seeking Medical Services. *Annals of plastic surgery*. 2018;81(6):725-729. doi:[10.1097/SAP.0000000000001582](https://doi.org/10.1097/SAP.0000000000001582)

136. Haupt C, Henke M, Kutschmar A, Hauser B, Baldinger S, Schreiber G. Antiandrogens or estradiol treatments or both during hormone replacement therapy in transitioning transgender women. *Cochrane Database of Systematic Reviews*. 2018;2018(10). doi:[10.1002/14651858.CD013138](https://doi.org/10.1002/14651858.CD013138)

137. Gilbert Paul A, Pass Lauren E, Keuroghlian Alex S, Greenfield Tom K, Reisner Sari L. Alcohol research with transgender populations: A systematic review and recommendations to strengthen future studies. *Drug and Alcohol Dependence*. 2018;186:138-146. doi:[10.1016/j.drugalcdep.2018.01.016](https://doi.org/10.1016/j.drugalcdep.2018.01.016)

138. Campbell Marianne, Hinton Jordan D. X, Anderson Joel R. A systematic review of the relationship between religion and attitudes toward transgender and gender-variant people. *International Journal of Transgenderism*. 2019;20(1):21-38. doi:[10.1080/15532739.2018.1545149](https://doi.org/10.1080/15532739.2018.1545149)

139. White Hughto JM, Reisner SL. A Systematic Review of the Effects of Hormone Therapy on Psychological Functioning and Quality of Life in Transgender Individuals. *Transgender health*. 2016;1(1):21-31. doi:[10.1089/trgh.2015.0008](https://doi.org/10.1089/trgh.2015.0008)

140. Valentine Sarah E, Shipherd Jillian C. A systematic review of social stress and mental health among transgender and gender non-conforming people in the United States. *Clinical Psychology Review*. 2018;66:24-38. doi:[10.1016/j.cpr.2018.03.003](https://doi.org/10.1016/j.cpr.2018.03.003)

141. Barone M, Cogliandro A, Di Stefano N, Tambone V, Persichetti P. A Systematic Review of Patient-Reported Outcome Measures Following Transsexual Surgery. *Aesthetic plastic surgery*. 2017;41(3):700-713. doi:[10.1007/s00266-017-0812-4](https://doi.org/10.1007/s00266-017-0812-4)

142. Frey JD, Poudrier G, Chiodo MV, Hazen A. A Systematic Review of Metoidioplasty and Radial Forearm Flap Phalloplasty in Female-to-male Transgender Genital Reconstruction: Is the “Ideal” Neophallus an Achievable Goal? *Plastic and reconstructive surgery Global open*. 2016;4(12):e1131. doi:[10.1097/GOX.0000000000001131](https://doi.org/10.1097/GOX.0000000000001131)

143. Glynn TR, van den Berg JJ. A Systematic Review of Interventions to Reduce Problematic Substance Use Among Transgender Individuals: A Call to Action. *Transgender health*. 2017;2(1):45-59. doi:[10.1089/trgh.2016.0037](https://doi.org/10.1089/trgh.2016.0037)

144. Shulman Grant P, Holt Natalie R, Hope Debra A, Mocarski Richard, Eyer Joshua, Woodruff Nathan. A review of contemporary assessment tools for use with transgender and gender nonconforming adults. *Psychology of Sexual Orientation and Gender Diversity*. 2017;4(3):304-313. doi:[10.1037/sgd0000233](https://doi.org/10.1037/sgd0000233)
